# Enhancing risk prediction for sudden cardiac death: quantitative late gadolinium enhancement analysis in hypertrophic cardiomyopathy

**DOI:** 10.1186/s13244-026-02385-3

**Published:** 2026-08-26

**Authors:** Lili Wang, Hongbo Zhang, Guanyu Lu, Junjie Zhou, Zhihui Lu, Lanling Wang, Chen Zhang, Xiaohai Ma, Shuang Li, Lei Zhao

**Affiliations:** 1 Department of Radiology, Beijing Anzhen Hospital, Capital Medical University, Beijing, China; 2 Department of Interventional Diagnosis and Treatment, Beijing Anzhen Hospital, Capital Medical University, Beijing, China

**Keywords:** Cardiac magnetic resonance, Hypertrophic cardiomyopathy, Late gadolinium enhancement quantification, Sudden cardiac death

## Abstract

**Objectives:**

To assess the prognostic value of multiple late gadolinium enhancement (LGE) quantification methods for sudden cardiac death (SCD) prediction in hypertrophic cardiomyopathy (HCM).

**Materials and methods:**

In this single-center retrospective study, 617 HCM patients who underwent cardiac magnetic resonance (CMR) examinations were consecutively enrolled. LGE was quantified using the *n*-standard deviation (SD) technique (with multiple thresholds) and the full width at half maximum method. “Gray zone” was defined as the myocardium with signal intensity between two predefined thresholds on LGE images. The primary outcome was SCD or aborted SCD.

**Results:**

Among 617 HCM patients, 424 (68.7%) were male, mean age was 49.1 ± 14.0 years, and LGE was identified in 438 (71.0%). During 67.9 ± 21.0 months of follow-up, 24 patients (3.9%) reached the primary endpoint. All dual-threshold gray zone measures and five single-threshold LGE quantifications (2–6 SDs) independently predicted SCD (HR range: 1.031–1.220, all adjusted *p* < 0.05). Gray zone (HR range: 1.091–1.220) showed stronger associations than single-threshold LGE measures (HR range: 1.031–1.046). Both approaches improved discrimination beyond the composite American Heart Association (AHA)/American College of Cardiology (ACC) model, with increased net reclassification improvement (0.321 and 0.397, respectively) and comparable C-statistics (0.794–0.871 vs 0.794–0.898, *p* = 0.155).

**Conclusion:**

While conventional single-threshold LGE quantification predicts SCD in HCM, dual-threshold gray-zone analysis demonstrates stronger associations. Both approaches provide incremental prognostic value beyond the AHA/ACC guideline-based risk model. These findings underscore the importance of standardizing LGE quantification to improve SCD risk stratification in patients with HCM.

**Key Points:**

- **Question:** The optimal LGE quantification approach for SCD risk stratification in HCM remains uncertain.
- **Findings:** Both single- and dual-threshold LGE quantification independently predict SCD in patients with HCM, while dual-threshold gray zone metrics demonstrate stronger prognostic associations.
- **Critical Relevance:** By systematically comparing multiple LGE quantification approaches, this study highlights the impact of methodological variability on SCD risk stratification in HCM, underscores the need for standardized LGE assessment, and informs clinical radiology practice.

**Graphical Abstract:**

**Figure:**
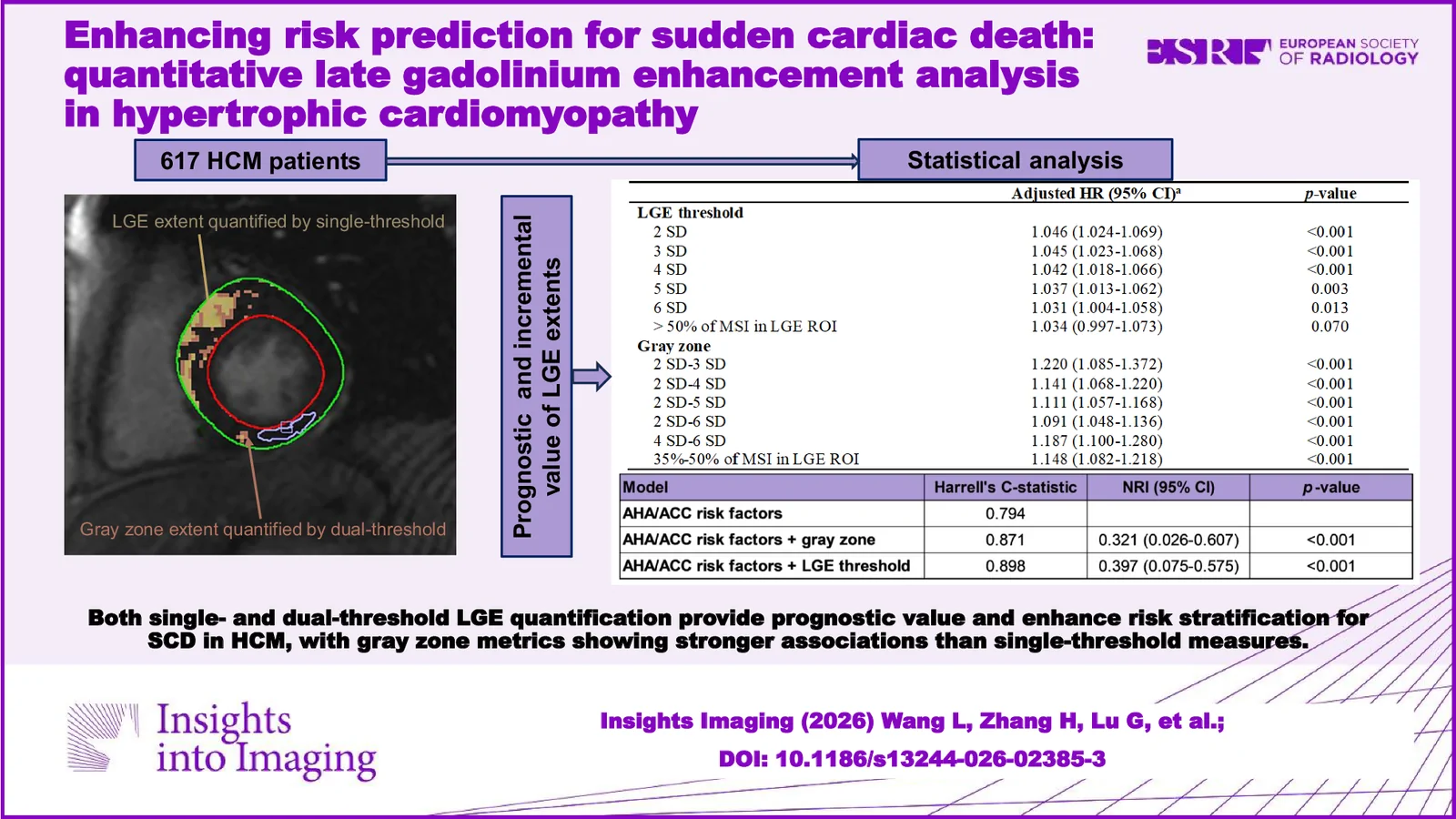

## Introduction

Hypertrophic cardiomyopathy (HCM), characterized by left ventricular (LV) hypertrophy unexplained by abnormal loading conditions, is the most common monogenic heart disease with a global prevalence of 0.2%–0.5% worldwide [1–3]. HCM is also a leading cause of sudden cardiac death (SCD) in young and otherwise healthy individuals [3–5]. While implantable cardioverter defibrillator (ICD) therapy is the most effective strategy for SCD prevention, identifying HCM patients who will benefit from ICD implantation remains challenging but crucial [3, 5]. Late gadolinium enhancement (LGE) cardiac magnetic resonance (CMR) enables noninvasive assessment of myocardial fibrosis, a pathophysiological hallmark of HCM. Multiple studies employing various LGE quantification methods have shown that LGE is an independent predictor of SCD in HCM [6–9]. Nowadays, extensive LGE (≥ 15% of the LV mass) has been incorporated as an SCD risk marker in the 2024 American Heart Association (AHA)/American College of Cardiology (ACC) and 2023 European Society of Cardiology (ESC) guidelines for HCM [4, 10]. However, no standardized LGE quantification method has been established for SCD risk stratification in HCM. Furthermore, the extent of “gray zone”, defined by intermediate signal intensity (SI) on LGE images and quantified using dual-threshold techniques, has demonstrated predictive value for SCD-related events both in ischemic cardiomyopathy (ICM) and non-ischemic cardiomyopathy (NICM) [11–15]. Multiple studies have reported that the gray zone extent was more strongly associated with SCD than the LGE extent conventionally quantified by a single threshold [11, 12, 14]. Nevertheless, it has not yet been exclusively investigated in HCM. Therefore, this study aimed to evaluate the prognostic value of various LGE quantification methods, including conventional single-threshold methods and dual-threshold approaches quantifying the gray zone, for SCD prediction in patients with HCM.

## Methods

### Study population

**Fig. 1:**
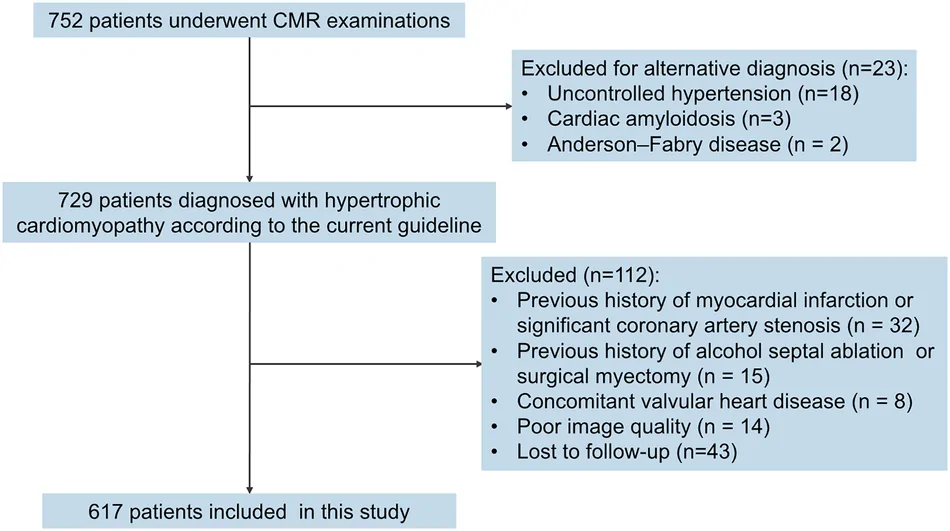
Study cohort. Flowchart of patient selection process according to inclusion and exclusion criteria. CMR, cardiac magnetic resonance

This retrospective study initially included 752 consecutive patients with suspected HCM (≥ 18 years of age) who underwent CMR examinations at our center between September 2015 and December 2021. HCM was diagnosed according to the AHA/ACC and ESC definitions of HCM [4, 10] based on increased LV wall thickness of (≥ 15 mm or 13–14 mm with family history) anywhere at end-diastole that cannot be attributed to any other abnormal loading conditions. The exclusion criteria were as follows: (1) uncontrolled hypertension (*n* = 18) and HCM phenocopies, including cardiac amyloidosis (*n* = 3) and Anderson–Fabry disease (*n* = 2); (2) previous history of myocardial infarction or significant coronary artery stenosis (*n* = 32); (3) previous history of alcohol septal ablation or surgical myectomy (*n* = 15); (4) concomitant valvular heart disease (*n* = 8); (5) poor image quality (*n* = 14); (6) lost to follow-up (*n* = 43). The study flowchart is presented in Fig. 1.

Demographic characteristics and medical history were obtained from an electronic medical record. History of atrial fibrillation (AF) and non-sustained ventricular tachycardia (NSVT) (≥ 3 consecutive ventricular beats at a rate of ≥ 120 bpm and < 30 s in duration) was identified from medical records, electrocardiograms, or 24 h Holter [10]. No patient had undergone ICD implantation before CMR examination. This study was approved by the Institutional Review Board of Beijing Anzhen Hospital (No.: 2025186x) and complied with the Declaration of Helsinki. Written informed consent was waived due to the retrospective nature of the study.

### CMR imaging

Image acquisition and parameters are detailed in the Supplemental Methods and Supplemental Table 1. Routine software updates occurred without affecting LGE quantification, and no major protocol or reconstruction changes were made.

**Fig. 2:**
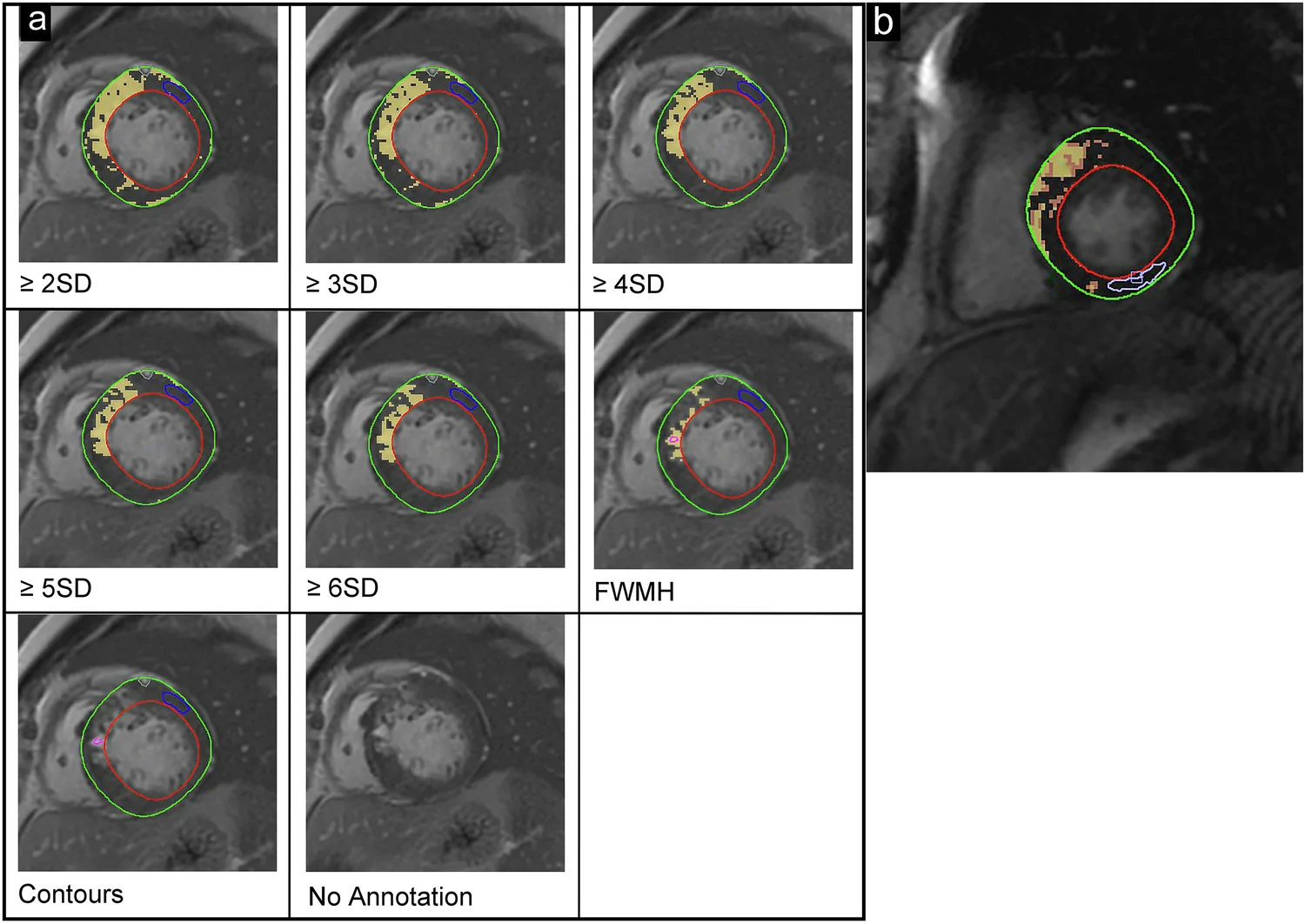
Methods of LGE quantification in HCM. **a** Representative short-axis LGE images demonstrating myocardial contouring using various single-threshold quantification methods. A small branch of the left anterior descending coronary artery was manually excluded (gray). ROIs include the area of maximal SI (pink) and normal reference myocardium (blue). High LGE-SI areas (yellow shading) are identified based on predefined SI thresholds. **b** The high LGE-SI area (yellow shading) and intermediate LGE-SI region (red shading) are semiautomatically delineated based on SI thresholds between 4 SD and 6 SD above the normal reference myocardium (blue). Endocardial (red) and epicardial (green) contours are manually delineated in (**a**, **b**). LGE, late gadolinium enhancement; HCM, hypertrophic cardiomyopathy; ROI, region of interest; SI, signal intensity; SD, standard deviation; FWHM, full width at half maximum

Quantitative analysis of LV function, structure, and LGE was independently performed by two blinded radiologists (L.W., 2 years of CMR experience; G.L., 6 years of CMR experience) using CVI42 software (version 5.17, Circle Cardiovascular Imaging). LGE presence was visually confirmed by both readers, with discrepancies resolved by a senior radiologist (L.Z., with 15 years of CMR experience). Quantification was restricted to enhancement confirmed on both short-axis and at least one orthogonal long-axis view. LGE was quantified using two semi-automated techniques, as demonstrated in Fig. 2: (1) the n-standard deviation (SD) technique, defining LGE-positive as voxels with SI ≥ n SD above the mean SI of the normal region of interest (ROI) (*n* = 2–6) [16]; (2) the full width at half maximum (FWHM) technique, defining LGE-positive as voxels with SI ≥ 50% of maximal SI within the LGE ROI [16]. Gray zones were calculated as the differences between: (1) 2 SD and 3 SD, (2) 2 SD and 4 SD, (3) 2 SD and 5 SD, (4) 2 SD and 6 SD, (5) 4 SD and 6 SD, and (6) 35% and 50% of maximal SI in the LGE ROI. All contours were semi-automatically generated and manually refined, and regions judged to be artifacts by readers were manually excluded. LGE extent was expressed as a percentage of LV mass.

### Clinical follow-up

The primary endpoint was a composite of SCD or aborted SCD. SCD was defined as sudden death witnessed or occurring within 1 h of new cardiac symptoms, or nocturnal death without prior progressive cardiac deterioration [17]. Aborted SCD was identified as an appropriate ICD shock for ventricular arrhythmia, an episode of ventricular fibrillation or spontaneous sustained ventricular tachycardia causing hemodynamic instability and requiring cardioversion. Aborted SCD was considered equivalent to SCD during follow-up [18]. Clinical follow-up was conducted by two radiologists (H.Z., with 5 years of CMR experience; J.Z., with 1 year of CMR experience) blinded to CMR data, using medical records, routine clinical visits, and telephone interviews. Follow-up duration was defined from the date of CMR examination to endpoint occurrence or last contact.

### Statistical analysis

Continuous data were reported as means ± SD or median (interquartile range); categorical data as n (%). Normality was assessed using the Kolmogorov–Smirnov test, supplemented by visual inspection of histograms and normal Q–Q plots. Comparisons between two groups for continuous variables were made using either Student’s *t*-test or Mann–Whitney *U*-test. Categorical variables were compared using the Chi-square test or Fisher’s exact test. Associations between LGE extent and AHA/ACC SCD risk markers in HCM were assessed by Spearman rank correlation.

Multivariable Cox regression analyses were performed separately to assess the independent prognostic value of each LGE quantification method, adjusting for variables identified in univariable analyses (*p* < 0.05). These covariates were combined into a composite continuous variable by assigning one point per categorical variable and summing the points [19]. Receiver operating curve (ROC) was performed on two methods: the single-threshold approach with the highest hazard ratio (HR) and the dual-threshold method with the highest HR. Optimal cut-offs were defined by the Youden index, and areas under the curve (AUCs) were compared using DeLong’s test. Event-free survival curves were compared using the Kaplan-Meier method and log-rank test.

Incremental predictive value was assessed using the category-free net reclassification improvement (NRI) by adding LGE extent to the established AHA/ACC model. Family history of SCD, LV maximal wall thickness ≥ 30 mm, unexplained syncope, apical aneurysm, LV ejection fraction (LVEF) < 50%, and NSVT were each treated as binary variables and summed to generate a count-based continuous variable referred to as “AHA/ACC risk factors” (0–6), representing the number of individual risk factors present in each patient. Model performance was evaluated using Harrell’s C-statistic, with differences assessed by DeLong’s test. The bootstrapping method with 1000 resamples was performed for internal validation of the HR and *C*-index. Multiple comparisons were corrected using the Benjamini-Hochberg false discovery rate (BH-FDR).

Intra- and interobserver reproducibility for LGE quantification were detailed in the Supplemental methods.

All statistical analyses were performed using SPSS version 24.0 (SPSS Inc.) and R (version 4.5.1, R Foundation for Statistical Computing). A two-tailed *p*-value < 0.05 was considered statistically significant.

## Results

### Study population

The final cohort included 617 HCM patients. Among them, 424 (68.7%) patients were male, and the mean age was 49.1 ± 14.0 years. Family history of SCD, New York Heart Association (NYHA) class III/IV, NSVT, unexplained syncope, and AF were present in 3.7%, 9.1%, 6.8%, 10.6%, and 12.0% of patients, respectively. CMR examinations were performed using Siemens (49.9%), Philips (27.4%), and GE (22.7%) scanners, respectively (Supplemental Table 2).

During a mean follow-up period of 67.9 ± 21.0 months (range, 49.0–81.0 months), 40 (6.5%) patients received prophylactic ICD therapies for primary prevention of SCD, of whom 10 had appropriate ICD shocks. The primary endpoint occurred in 24 (3.9%) patients: 12 (1.9%) SCD and 12 (1.9%) aborted SCD (2 sustained ventricular tachycardia, 10 appropriate ICD shocks). Patients suffering from SCD more often had a family history of SCD, NYHA class III/IV, NSVT, AF, unexplained syncope, palpitations, shortness of breath, and β-blocker therapy (all *p* < 0.05). Baseline characteristics are detailed in Table 1.

**Table 1:** Baseline characteristics of study participants

	All patients (*n* = 617)	Patients without SCD end point (*n* = 593)	Patients with SCD end point (*n* = 24)	*p*-value
Age (years)	49.1 ± 14.0	49.2 ± 13.7	45.2 ± 20.7	0.35
Male sex, *n* (%)	424 (68.7)	411(69.3)	13 (54.2)	0.12
Body mass index (kg/m^2^)	26.0 ± 3.3	26.1 ± 3.3	25.0 ± 3.9	0.22
Hypertension, *n* (%)	251 (40.7)	242 (40.8)	9 (37.5)	0.75
Diabetes, *n* (%)	68 (11.0)	64 (10.8)	4 (16.7)	0.57
Hyperlipidaemia, *n* (%)	142 (23.0)	139 (23.4)	3 (12.5)	0.22
Family history of SCD, *n* (%)	23 (3.7)	19 (3.2)	4 (16.7)	0.01
NYHA class III/IV, *n* (%)	56 (9.1)	50 (8.4)	6 (25.0)	0.02
Non-sustained VT, *n* (%)	42 (6.8)	28 (4.7)	14 (58.3)	< 0.001
AF, *n* (%)	74 (12.0)	67 (11.3)	7 (29.2)	0.002
Syncope, *n* (%)	64 (10.6)	56 (9.6)	8 (34.8)	< 0.001
Palpitation, *n* (%)	207 (34.3)	193 (33.3)	14 (58.3)	0.01
Chest discomfort, *n* (%)	427 (69.8)	410 (69.7)	17 (70.8)	0.91
Shortness of breath, *n* (%)	224 (36.3)	208 (35.1)	16 (66.7)	0.002
β-blocker, *n* (%)	295 (47.9)	277 (46.8)	18 (75.0)	0.007
Calcium channel blockers, *n* (%)	174 (28.2)	164 (27.7)	10 (41.7)	0.14

Values are presented as *n* (%) or mean ± SD

*SCD* sudden cardiac death, *NYHA* New York Heart Association, *VT* ventricular tachycardia

### CMR findings and associations between LGE extents and SCD risk markers

As presented in Table 2, patients who experienced SCD had a higher LV maximal wall thickness (25.0 ± 5.2 vs 20.3 ± 5.4 mm, *p* < 0.001), lower stroke volume (72.5 ± 15.2 vs 88.0 ± 24.5, *p* < 0.001), and lower LVEF (54.3 ± 16.4 vs 62.1 ± 10.0, *p* = 0.03) compared to patients without SCD. Furthermore, SCD patients exhibited a higher prevalence of LVEF < 50% (41.7% vs 9.4%, *p* < 0.001), apical aneurysm (33.3% vs 6.2%, *p* < 0.001), and positive LGE (100.0% vs 69.8%, *p* = 0.001). However, the prevalence of LV maximal wall thickness ≥ 30 mm did not differ significantly (*p* = 0.054). Additionally, LGE was present in 438 (71.0%) patients by visual assessment. In patients with positive LGE, those experiencing SCD consistently had greater LGE extents across methods (all *p* < 0.05), as demonstrated in Table 3.

**Table 2:** CMR imaging characteristics of study participants

	All patients (*n* = 617)	Patients without SCD end point (*n* = 593)	Patients with SCD end point (*n* = 24)	*p*-value
LA maximum volume (mL)	89.3 ± 35.5	89.2 ± 35.1	91.1 ± 46.1	0.84
LV end-diastolic diameter (mm)	45.8 ± 6.6	45.8 ± 6.5	45.0 ± 7.9	0.63
LV maximal wall thickness (mm)	20.5 ± 5.4	20.3 ± 5.4	25.0 ± 5.2	< 0.001
LV maximal wall thickness ≥ 30 mm, *n* (%)	35 (5.7)	31 (5.2)	4 (16.7)	0.054
LV mass index (g/m^2^)	48.1 ± 21.0	47.7 ± 20.5	59.4 ± 31.0	0.08
LV EDV index (mL/m^2^)	77.5 ± 20.8	77.4 ± 20.7	81.5 ± 24.5	0.42
LV ESV index (mL/m^2^)	30.3 ± 14.8	29.9 ± 14.2	39.7 ± 24.6	0.07
Stroke volume (mL)	87.4 ± 24.3	88.0 ± 24.5	72.5 ± 15.2	< 0.001
Cardiac index (L/min/m^2^)	3.2 ± 1.0	3.2 ± 1.0	2.9 ± 0.9	0.12
LV ejection fraction (%)	61.8 ± 10.4	62.1 ± 10.0	54.3 ± 16.4	0.03
LV ejection fraction < 50%, *n* (%)	66 (10.7)	56 (9.4)	10 (41.7)	< 0.001
LVOT obstruction, *n* (%)	238 (38.6)	228 (38.4)	10 (41.7)	0.75
Apical aneurysm, *n* (%)	45 (7.3)	37 (6.2)	8 (33.3)	< 0.001
LGE presence, *n* (%)	438 (71.0)	414 (69.8)	24 (100.0)	0.001

Values are presented as *n* (%) or mean ± SD

*SCD* sudden cardiac death, *LA* left atrial, *LV* left ventricular, *EDV* end-diastolic volume, *ESV* end-systolic volume, *LVOT* left ventricular outflow tract, *LGE* late gadolinium enhancement

**Table 3:** LGE extents quantified by different methods in study participants with positive LGE

	All patients (*n* = 438)	Patients without SCD end point (*n* = 414)	Patients with SCD end point (*n* = 24)	*p*-value
LGE threshold (%)				
2 SD	24.1 [14.4–39.9]	23.4 [13.7–37.8]	50.9 [41.6–61.6]	< 0.001
3 SD	16.4 [9.1–30.4]	15.8 [8.5–28.7]	44.2 [32.1–50.5]	< 0.001
4 SD	10.9 [5.5–23.1]	10.6 [5.2–21.7]	34.4 [23.9–41.8]	< 0.001
5 SD	7.5 [3.4–17.6]	7.0 [3.2–16.9]	27.7 [17.2–35.1]	< 0.001
6 SD	5.3 [2.1–13.7]	4.9 [2.0–12.8]	22.4 [12.0–29.0]	< 0.001
> 50% of MSI in LGE ROI	1.1 [0–4.1]	0.9 [0–3.5]	9.4 [2.9–18.9]	< 0.001
Gray zone (%)				
2 SD–3 SD	7.1 [4.9–9.5]	7.0 [4.8–9.4]	9.1 [7.6–11.3]	0.002
2 SD–4 SD	12.3 [8.2–16.5]	12.1 [8.0–16.0]	16.7 [13.4–22.2]	< 0.001
2 SD–5 SD	15.9 [10.1–21.7]	15.6 [9.7–21.1]	23.4 [17.8–30.4]	< 0.001
2 SD–6 SD	18.6 [11.3–25.6]	18.2 [11.1–24.7]	28.7 [21.0–38.2]	< 0.001
4 SD–6 SD	5.8 [3.2–9.1]	5.5 [3.1–8.7]	11.5 [6.9–16.5]	< 0.001
35%–50% of MSI in LGE ROI	2.0 [0–6.9]	1.8 [0–6.1]	11.3 [6.7–19.3]	< 0.001

*LGE* late gadolinium enhancement, *SCD* sudden cardiac death, *SD* standard deviation, *MSI* maximal signal intensity, *ROI* region of interest

The prevalence of unexplained syncope, family history of SCD, NSVT, apical aneurysm, LV maximal wall thickness ≥ 30 mm and LVEF < 50% were grouped, while all LGE extents evaluated by different quantification methods formed a separate group. As shown in Fig. S1, no statistically significant correlation was observed between these two groups (ρ range: −0.01 to 0.28). In contrast, a moderate to strong correlation was found among LGE quantification methods (ρ range: 0.58–1.00).

### Associations between LGE extents and the primary outcome

Unexplained syncope, family history of SCD, NSVT, NYHA class III/IV, AF, LV maximal wall thickness ≥ 30 mm, LV mass index, LVEF < 50%, and apical aneurysm were associated with SCD in univariable analysis (HR range = 1.02–23.29, all *p* < 0.05) (Supplemental Table 3). As shown in Table 4, LGE extents quantified using a single threshold (2–6 SDs) and all gray zone extents were independently associated with SCD after adjusting for a composite variable (HR range = 1.031–1.220, all adjusted *p* < 0.05). Furthermore, gray zone measures yielded higher HRs for SCD than single-threshold quantifications (1.091–1.220 vs 1.031–1.046).

**Table 4:** Multivariable Cox regression analysis for different LGE quantification methods in predicting SCD

	Adjusted HR (95% CI)^a^	*p*-value
LGE threshold		
2 SD	1.046 (1.024–1.069)	< 0.001
3 SD	1.045 (1.023–1.068)	< 0.001
4 SD	1.042 (1.018–1.066)	< 0.001
5 SD	1.037 (1.013–1.062)	0.003
6 SD	1.031 (1.004–1.058)	0.013
> 50% of MSI in LGE ROI	1.034 (0.997–1.073)	0.070
Gray zone		
2 SD–3 SD	1.220 (1.085–1.372)	< 0.001
2 SD–4 SD	1.141 (1.068–1.220)	< 0.001
2 SD–5 SD	1.111 (1.057–1.168)	< 0.001
2 SD–6 SD	1.091 (1.048–1.136)	< 0.001
4 SD–6 SD	1.187 (1.100–1.280)	< 0.001
35%–50% of MSI in LGE ROI	1.148 (1.082–1.218)	< 0.001

*LGE* late gadolinium enhancement, *HR* hazard ratio, *CI* confidence interval, *SD* standard deviation, *MSI* maximal signal intensity, *ROI* region of interest

^a^The multivariable model was adjusted for a composite risk factor

**Fig. 3:**
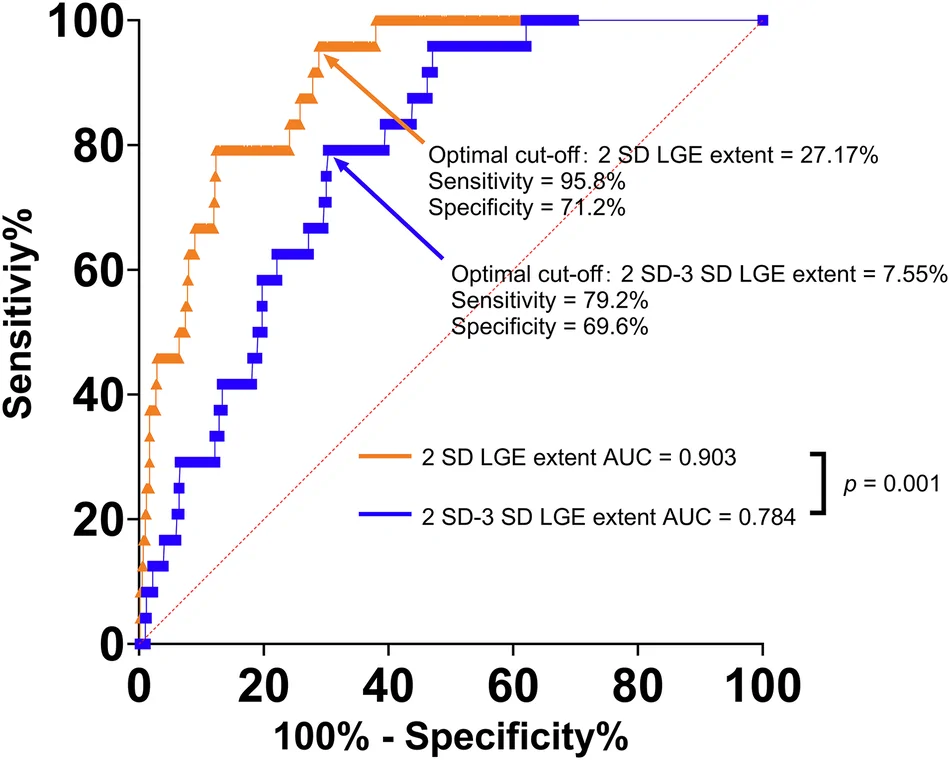
ROC curves of LGE quantification by 2 SD and 2 SD–3 SD methods for SCD. Arrows indicate optimal cut-offs. ROC, receiver operating characteristic; LGE, late gadolinium enhancement; SD, standard deviation; SCD, sudden cardiac death; AUC, area under the curve

**Fig. 4:**
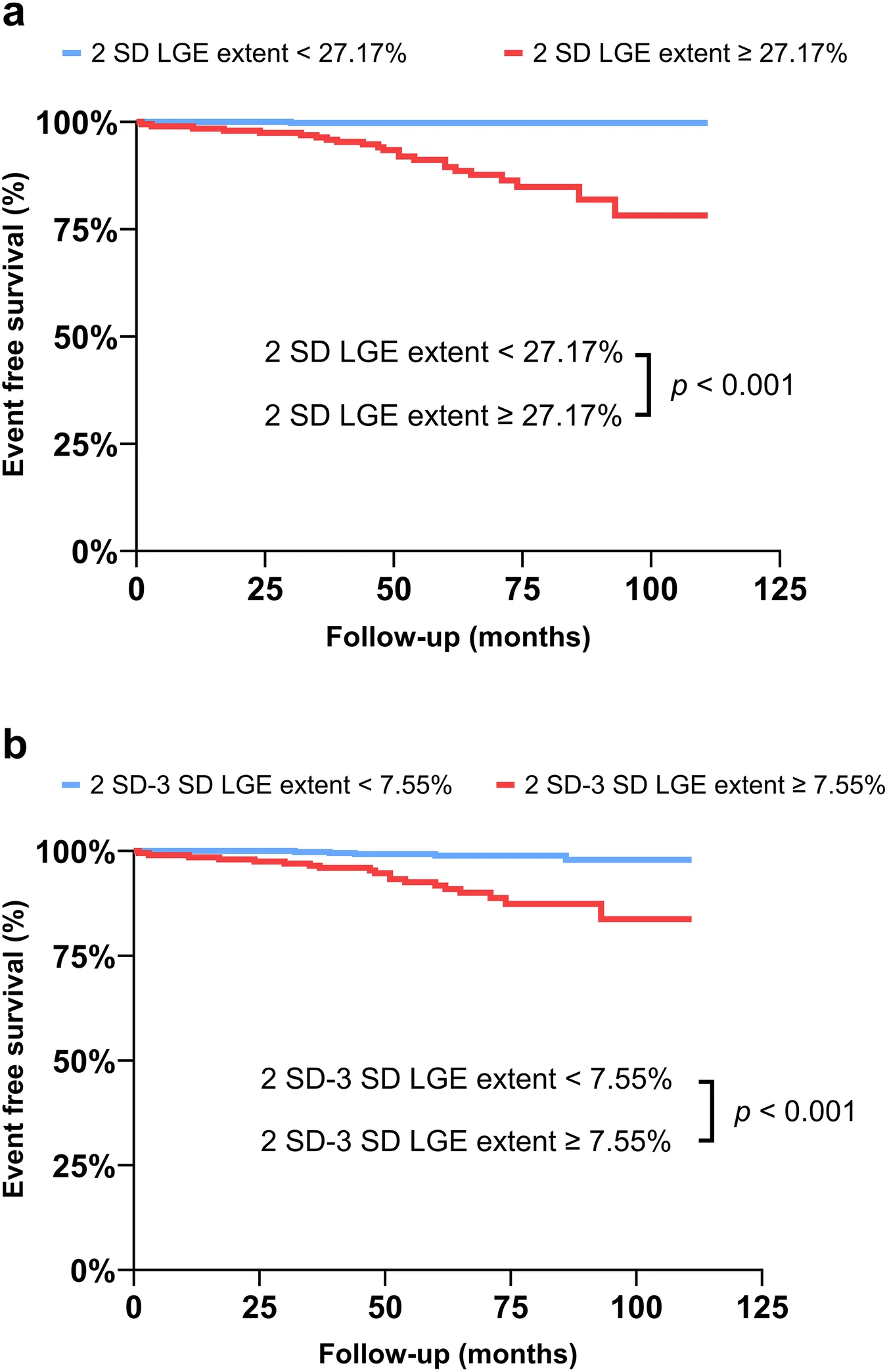
Kaplan–Meier analysis for SCD between groups with different LGE extents. Curves showing the incidence of SCD stratified by the extent of LGE quantified using the 2 SD method (**a**) and the 2 SD–3 SD method (**b**). SCD, sudden cardiac death; LGE, late gadolinium enhancement; SD, standard deviation

From the ROC analysis, the LGE extent defined by the 2 SD method, the single-threshold technique with the highest HR (HR: 1.046 [95% CI: 1.024–1.069]; *p* < 0.001) and the 2 SD–3 SD approach (HR: 1.220 [95% CI: 1.085–1.372]; *p* < 0.001), the top-performing dual-threshold technique, yielded optimal cut-off values of 27.17% and 7.55%, respectively (Fig. 3). The corresponding AUCs were 0.903 and 0.784, with a statistically significant difference between the two (*p* = 0.001). Kaplan–Meier analysis demonstrated significantly higher SCD risk in patients with LGE above each cut-off (all *p* < 0.001), as shown in Fig. 4.

### Incremental prognostic value of LGE extents for SCD

**Fig. 5:**
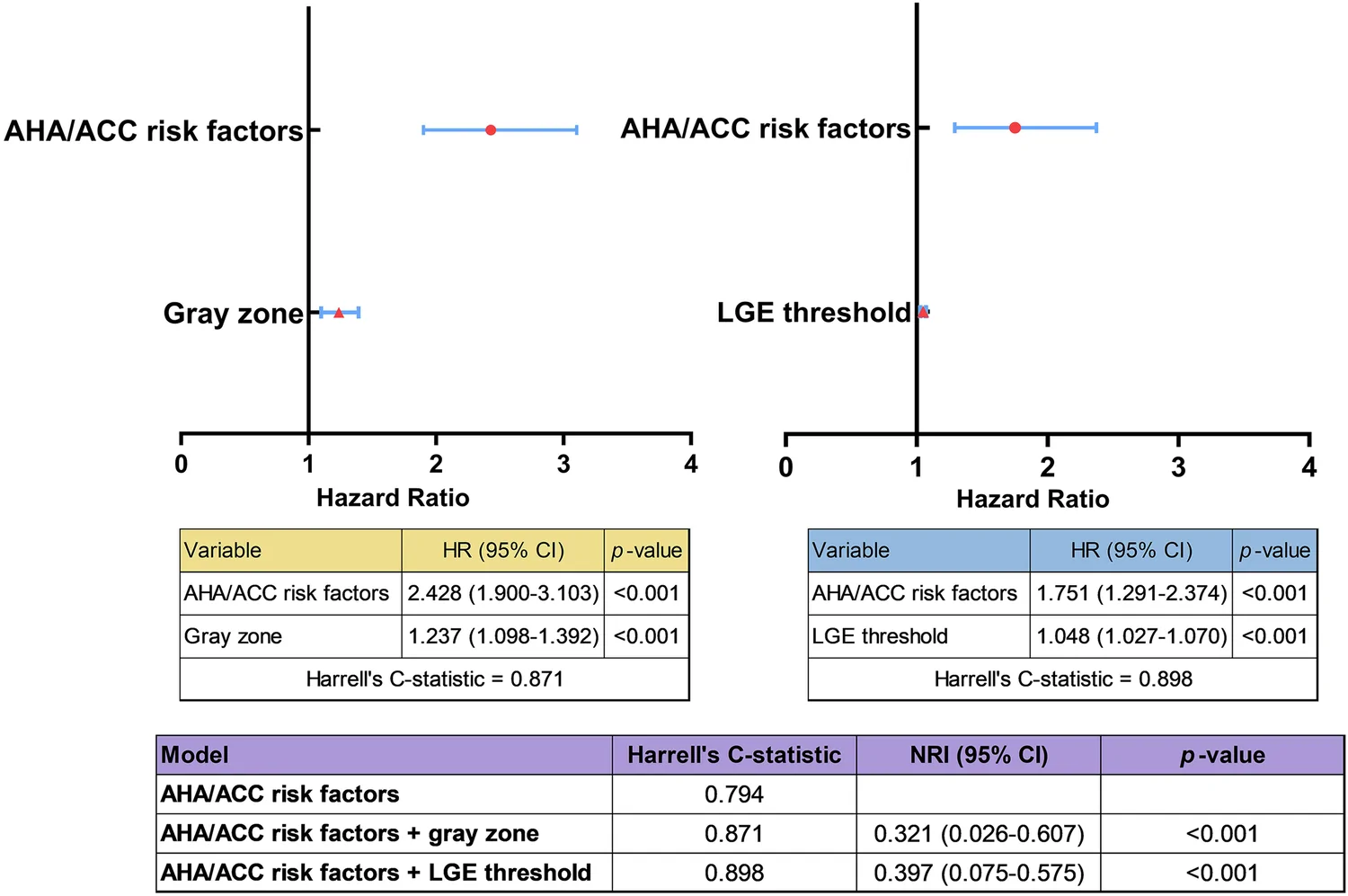
Multivariable Cox regression and NRI analyses for SCD with subsequent addition of the LGE quantification. (Top) Forest plots from multivariable Cox models evaluating the association of a composite AHA/ACC risk factor variable with either the gray zone extent (left) or a single-threshold LGE extent (right). (Middle) HRs and model performance for the composite AHA/ACC risk factors with the addition of gray zone or LGE threshold. Harrell’s C-statistics were comparable between the two models (0.871 vs 0.898; *p* = 0.155). (Bottom) Model discrimination and risk reclassification analysis for SCD prediction. NRI, net reclassification improvement; SCD, sudden cardiac death; LGE, late gadolinium enhancement; HR, hazard ratio; CI, confidence interval; AHA, American Heart Association; ACC, American College of Cardiology

After adjusting for a composite AHA/ACC risk factor, LGE extents quantified by 2 SD and 2 SD-3 SD remained independently associated with SCD (HR: 1.048 [95% CI: 1.027–1.070], *p* < 0.001; HR: 1.237 [95% CI: 1.098–1.392], *p* < 0.001, respectively) (Fig. 5). Incorporation of LGE extent, whether quantified using single- or dual-threshold method, significantly improved the discrimination of the AHA/ACC risk factor model, with NRIs of 0.397 [95% CI: 0.075–0.575] and 0.321 [95% CI: 0.026–0.607], respectively. Model performance was comparable with the inclusion of either a single-threshold LGE extent or the gray zone extent alone, as evidenced by *C*-indices of 0.794–0.898 and 0.794–0.871, respectively (*p* = 0.155).

### Reproducibility

Reproducibility for all LGE quantification methods was summarized in Table 5. The FWHM technique (50% maximal SI within the LGE ROI) demonstrated the highest reproducibility, with intraobserver and interobserver intraclass correlation coefficients (ICCs) of 0.92 and 0.90, respectively. The 2 SD threshold demonstrated good reproducibility (ICC: 0.81 and 0.76), whereas the gray zone (2 SD–3 SD) showed moderate to good reproducibility (ICC: 0.80 and 0.73).

**Table 5:** Intraobserver and interobserver reproducibility of LGE quantification

	Intraobserver ICC	*p*-value	Interobserver ICC	*p*-value
LGE threshold				
2 SD	0.81	< 0.001	0.76	< 0.001
3 SD	0.83	< 0.001	0.78	< 0.001
4 SD	0.85	< 0.001	0.81	< 0.001
5 SD	0.86	< 0.001	0.82	< 0.001
6 SD	0.88	< 0.001	0.85	< 0.001
> 50% of MSI in LGE ROI	0.92	< 0.001	0.90	< 0.001
Gray zone				
2 SD–3 SD	0.80	0.048	0.73	< 0.001
2 SD–4 SD	0.82	0.006	0.77	< 0.001
2 SD–5 SD	0.84	0.002	0.78	< 0.001
2 SD–6 SD	0.84	0.001	0.81	< 0.001
4 SD–6 SD	0.85	< 0.001	0.81	< 0.001
35%–50% of MSI in LGE ROI	0.90	< 0.001	0.90	< 0.001

*LGE* late gadolinium enhancement, *SD* standard deviation, *MSI* maximal signal intensity, *ROI* region of interest

## Discussion

In this study, we systematically evaluated the prognostic value of multiple LGE quantification methods for SCD prediction in patients with HCM. The key findings can be summarized as follows: (1) LGE extents quantified by single-threshold techniques (2–6 SD methods) and dual-threshold methods (all gray zone extents) independently predicted SCD after FDR correction; (2) gray zone extent demonstrated a stronger association with SCD than LGE extent defined by a single threshold, as assessed by HRs; and (3) incorporation of LGE extent into the composite AHA/ACC risk factor model improved risk discrimination, with comparable C-statistics between single- and dual-threshold LGE quantifications.

### Prognostic value of LGE quantification methods for SCD prediction

Current 2024 AHA/ACC and 2023 ESC guidelines have incorporated extensive LGE into SCD risk markers in HCM but do not specify a preferred quantification method [4, 10]. Multiple LGE quantification methods, including single- and dual-threshold approaches, have been applied across studies in ICM and NICM [13, 14, 20–22]. Variability among LGE quantification techniques results in substantial differences in reported LGE extent [13, 14, 20–22], potentially affecting the accuracy and comparability of SCD risk stratification. Therefore, standardization of LGE assessment is critical for cross-study consistency and clinical decision-making.

In our HCM cohort, LGE extent defined by n-SD thresholds ranging from 2 to 6 SD remained independently associated with SCD after FDR correction, consistent with previous studies [6, 8, 19, 23]. Notably, HRs for SCD progressively decreased with increasing threshold stringency, aligning with findings of a recent study using a composite endpoint of appropriate ICD shock and all-cause mortality [14]. However, LGE extent quantified by the FWHM technique did not retain an independent prognostic role after adjustment, consistent with findings by Ismail et al [24].

In accordance with previous studies [11, 12, 14], we found that all gray zone quantifications were independently associated with SCD in HCM after FDR correction. In head-to-head comparisons, the gray zone extent showed a stronger association with SCD than LGE extent measured by single-threshold methods, consistent with previous studies [11, 12, 14]. However, the single-threshold method demonstrated superior discriminative ability compared with gray-zone quantification when evaluated alone (AUC: 0.903 vs 0.784, *p* = 0.001). When LGE extents were incorporated into the AHA/ACC risk factor model, overall model discrimination improved, highlighting the incremental prognostic value of LGE beyond established risk markers. Notably, model discrimination was similar regardless of whether LGE was quantified by single- or dual-threshold approaches (C-statistic: 0.898 vs 0.871, *p* = 0.155). This observation parallels findings from a prospective ICM cohort [11], in which predictive models performed similarly whether they included core-infarct mass, peri-infarct mass, or both. In ICM, these peri-infarct “border zones” [11], defined as intermediate-signal areas adjacent to dense infarct cores, have been confirmed to consist of a heterogeneous mixture of viable myocytes and fibrotic scar tissue by histological evidence [25]. Our study suggests that a similar mechanism may apply in HCM. Specifically, gray zones in HCM may represent components of myocardial fibrosis, which is often unevenly distributed around myocytes [26]. This heterogeneous fibrosis and irregular shape could be more arrhythmogenic than homogeneous fibrosis [27]. Maladaptive remodeling of this fibrotic tissue could ultimately lead to irreversible dysfunction such as SCD [26]. However, direct histological validation of this mechanism in HCM is challenging, as correlating LGE with myocardial pathology would require matching imaging to post-mortem tissue from the same individuals, which is rarely feasible. This limitation underscores the need for future studies to verify the histopathologic substrate of LGE in HCM and further elucidate the mechanisms leading to SCD.

### Limitations

This study has several limitations. First, its retrospective, single-center design at a tertiary referral institution may have introduced referral bias and limited generalizability. Second, because LV maximal outflow tract gradient was unavailable, the ESC Risk-SCD score could not be calculated. Third, LGE quantification was performed using two-dimensional, breath-hold imaging with relatively thick slices (6–8 mm), future studies using three-dimensional may improve coverage and spatial resolution. Fourth, differences in contrast agent type and dosing may have influenced LGE quantification. Fifth, despite BH-FDR correction and internal bootstrap validation, comparisons across multiple LGE quantifications and the data-derived cutoffs remain exploratory, requiring external validation before routine clinical implementation. Finally, the low incidence of SCD events, consistent with prior HCM cohorts [3, 19, 28], may limit statistical power and preclude reliable assessment of vendor-specific performance. Larger multicenter studies are therefore warranted.

## Conclusions

Single- and dual-threshold LGE measures were independently associated with SCD in HCM, with stronger associations observed for dual-threshold approaches. Both methods improved discrimination beyond the AHA/ACC risk factor model. These findings support standardizing LGE quantification to ensure reproducible and accurate SCD risk stratification in clinical practice and future research.

## Supplementary information

ELECTRONIC SUPPLEMENTARY MATERIAL

## Data Availability

The datasets generated and analyzed in this study are not publicly available due to patient privacy but may be made available in de-identified form to the corresponding author upon reasonable request and approval by the relevant ethics committee.

## Abbreviations

ACC: American College of Cardiology
AF: Atrial fibrillation
AHA: American Heart Association
AUC: Area under the curve
CI: Confidence interval
CMR: Cardiac magnetic resonance
ESC: European Society of Cardiology
FWHM: Full width at half maximum
HCM: Hypertrophic cardiomyopathy
HR: Hazard ratio
ICC: Intraclass correlation coefficient
ICD: Implantable cardioverter defibrillator
ICM: Ischemic cardiomyopathy
LGE: Late gadolinium enhancement
LV: Left ventricular
LVEF: Left ventricular ejection fraction
MSI: Maximal signal intensity
NICM: Non-ischemic cardiomyopathy
NRI: Net reclassification improvement
NYHA: New York Heart Association
NSVT: Non-sustained ventricular tachycardia
ROI: Region of interest
ROC: Receiver operating curve
SCD: Sudden cardiac death
SD: Standard deviation
SI: Signal intensity
