# Supplementary material for: Enhancing risk prediction for sudden cardiac death: quantitative late gadolinium enhancement analysis in hypertrophic cardiomyopathy
Source: Insights Imaging. 2026 Aug 26;17:210. doi: 10.1186/s13244-026-02385-3 (PMC13518643; doi:10.1186/s13244-026-02385-3)
Supplement: Supplementary file 1 [file 13244_2026_2385_MOESM1_ESM.pdf]

Enhancing Risk Prediction for Sudden Cardiac Death: Quantitative Late Gadolinium Enhancement Analysis in Hypertrophic Cardiomyopathy

Electronic Supplementary Materials

Supplemental methods

CMR image acquisition

All CMR examinations were performed on 3.0 T scanners (Discovery MR750, GE medical systems; MAGNETOM Verio, Siemens Healthineers; Ingenia, Philips) using standardized cardiac protocols with electrocardiographic gating and breath-holding. Standard CMR protocols mainly consisted of (1) Balanced steady-state free precession cine sequences were acquired in the LV long-axis (two-, three-, and four-chamber views) as well as in a stack of short-axis slices covering the entire left ventricle; (2) LGE images were performed by 2-dimensional segmented phase-sensitive inversion-recovery sequences in the same views as the cine images, approximately 10-15 min after the intravenous administration of either gadopentetate dimeglumine (0.2 mmol/kg; Magnevist, Bayer Healthcare Pharmaceuticals) or gadobutrol (0.1 mmol/kg; Gadavist, Bayer Healthcare Pharmaceuticals).

Intra- and inter-observer agreement

Intra- and inter-observer agreement for LGE quantification were assessed in 30 randomly selected cases. For intra-observer variability, measurements were repeated by the same observer with an interval of at least 1 month. For inter-observer variability, a second observer, blinded to the results of the first observer, independently performed the analysis. Reliability was evaluated using the intraclass correlation coefficient.

Supplemental Table 1 Detailed CMR scan acquisition parameters of three 3.0T MR scanners

	GE		Siemens		Philips	
	**Cine**	**LGE**	**Cine**	**LGE**	**Cine**	**LGE**
TR (ms)	3.6	6.2	3.1	4.1	3	6.1
TE (ms)	1.4	2.9	1.3	1.6	1.5	3
FA (°)	60	25	45	20	45	25
Receiver bandwidth (Hz/px)	1041.7	284.1	704	156	1268.9	147
GRAPPA Acceleration factor	2	2	2	2	2	2
FOV (mm^2)	380*380	380*380	276*340	350*284	270*270	350*350
In-plane resolution (mm^2)	2.0*1.7	1.7*2.0	1.8*1.4	1.9*1.4	1.8*1.8	1.6*1.9
Temporal resolution (ms)	86	149	40-50	140-150	50	154

Cine images include 2-chamber, 3-chamber, 4-chamber views at long-axis and short-axis images. GE refers Discovery MR750 3.0T, GE Medical Systems, Milwaukee, USA; Siemens refers MAGNETOM Verio 3.0T, Siemens Healthineers, Erlangen, Germany; Phillips refers to Ingenia 3.0T, Phillips, Best, Netherlands. CMR, cardiovascular magnetic resonance; MR, magnetic resonance; LGE, late gadolinium enhancement; TR, repetition time; TE, echo time; FA, flip angel; FOV, field of view.

Supplemental Table 2 Univariate Cox regression analysis for clinical and CMR variables and different LGE quantification methods in predicting SCD

	HR (95% CI)	*P* Value
**Clinical variables**		
Age (years)	0.98 (0.95-1.01)	0.15
Male sex	0.53 (0.24-1.18)	0.12
Syncope	4.86 (2.08-11.36)	<0.001
Family history of SCD	5.09 (1.74-14.90)	0.003
Non-sustained VT	23.29 (10.29-52.70)	<0.001
NYHA class III/IV	3.80 (1.50-9.64)	0.005
Atrial fibrillation	3.82 (1.65-8.87)	0.002
**CMR variables**		
LA maximum volume (mL)	1.00 (0.99-1.01)	0.66
LV end-diastolic diameter (mm)	0.99 (0.93-1.05)	0.66
LV maximal wall thickness (mm)	1.08 (1.05-1.12)	<0.001
LV maximal wall thickness ≥30 mm	3.83 (1.31-11.25)	0.01
LV mass index (g/m2)	1.02 (1.01-1.03)	0.001
LV EDV index (mL/m2)	1.01 (0.99-1.03)	0.25
Cardiac index (L/min/m2)	0.74 (0.46-1.17)	0.19
LV ejection fraction (%)	0.95 (0.93-0.98)	<0.001
LV ejection fraction <50%	6.25 (2.77-14.07)	<0.001
LVOT obstruction	1.24 (0.55-2.79)	0.61
Apical aneurysm	6.48 (2.77-15.17)	<0.001
**Different LGE quantification methods**		
2 SD	1.07 (1.05-1.09)	<0.001
3 SD	1.09 (1.05-1.09)	<0.001
4 SD	1.07 (1.05-1.09)	<0.001
5 SD	1.07 (1.05-1.09)	<0.001
6 SD	1.07 (1.05-1.09)	<0.001
> 50% of MSI in LGE ROI	1.10 (1.07-1.13)	<0.001
2 SD-3 SD	1.28 (1.16-1.42)	<0.001
2 SD-4 SD	1.18 (1.11-1.25)	<0.001
2 SD-5 SD	1.15 (1.09-1.20)	<0.001
2 SD-6 SD	1.13 (1.09-1.17)	<0.001
4 SD-6 SD	1.32 (1.22-1.42)	<0.001
35%-50% of MSI in LGE ROI	1.22 (1.17-1.28)	<0.001

CMR, cardiac magnetic resonance; LGE, late gadolinium enhancement; SCD, sudden cardiac death; HR, hazard ratio; CI, confidence interval; VT, ventricular tachycardia; NYHA, New York Heart Association; LA, left atrial; LV, left ventricular; EDV, end-diastolic volume; LVOT, left ventricular outflow tract; SD, standard deviation; MSI, maximum pixel signal intensity; ROI, region of interest.

Figure S1 Associations between different extents of LGE and AHA/ACC risk markers for SCD

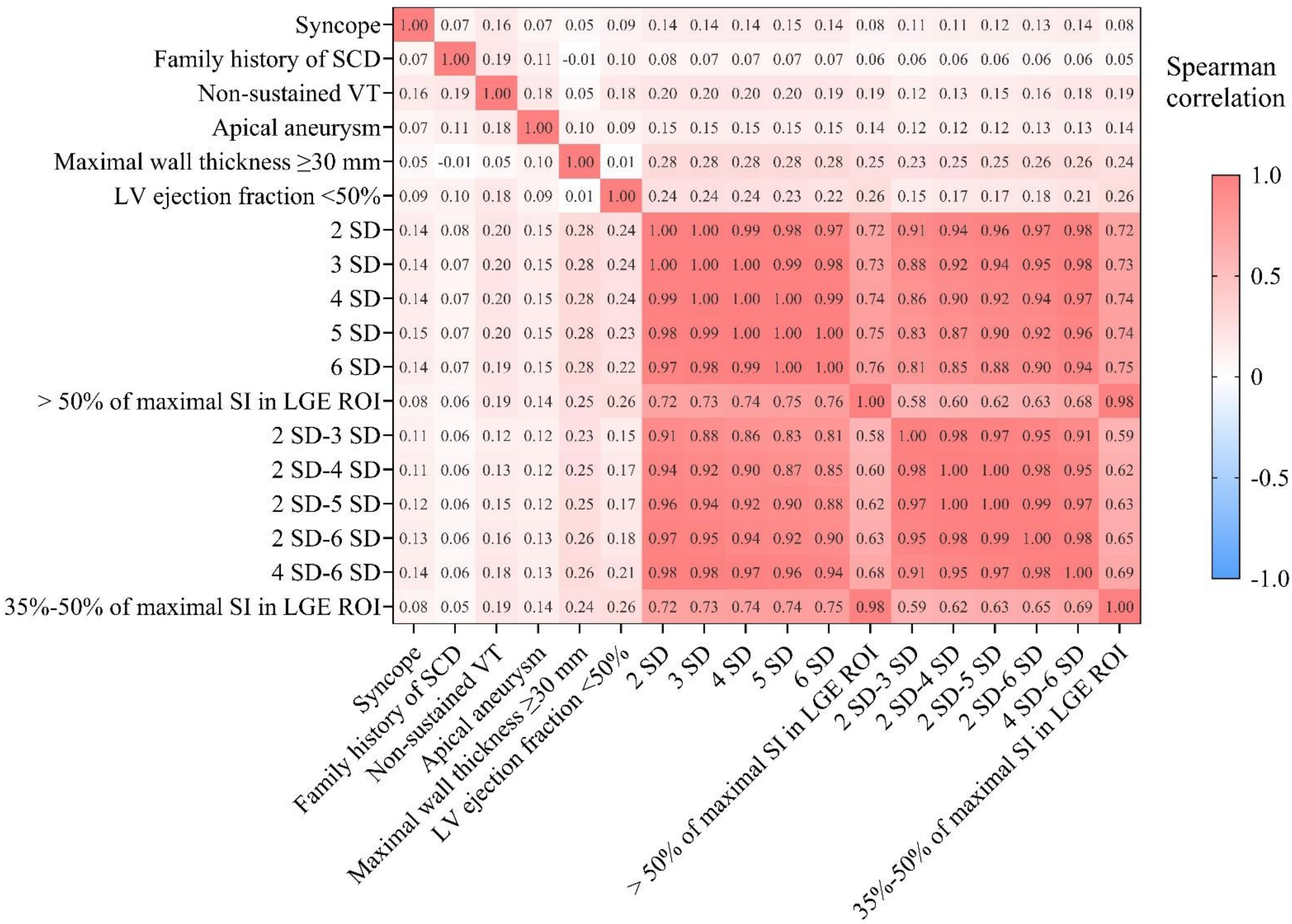

LGE: late gadolinium enhancement; AHA, American Heart Association; ACC, American College of Cardiology; SCD, sudden cardiac death; VT, ventricular tachycardia; LV, left ventricular; SD, standard deviation; MSI, maximum pixel signal intensity; ROI, region of interest.
